# Osteopontin promoter polymorphisms and risk of urolithiasis: a candidate gene association and meta-analysis study

**DOI:** 10.1186/s12881-020-01101-2

**Published:** 2020-08-25

**Authors:** Ali Amar, Ayesha Afzal, Athar Hameed, Mumtaz Ahmad, Abdul Rafay Khan, Humaira Najma, Aiysha Abid, Shagufta Khaliq

**Affiliations:** 1 Department of Human Genetics and Molecular Biology, University of Health Sciences, Khayaban-e-jamia Punjab, Lahore, Punjab 54600 Pakistan; 2 Department of Urology, Fatima Jinnah Medical University, Lahore, Punjab Pakistan; 3 Centre for Human Genetics and Molecular Medicine, Sindh Institute of Urology and Transplantation, Karachi, Pakistan

**Keywords:** Candidate Gene Association study, Meta-analysis, Pakistan, Renal calculi, SPP1, SNP

## Abstract

**Background:**

Urolithiasis is a worldwide urological problem with significant contribution of genetic factors. Pakistan, which resides within the Afro-Asian stone belt, has a high reported prevalence (12%) of urolithiasis. Osteopontin (SPP1) is a urinary macromolecule with a suggested critical role in modulating renal stone formation, genetic polymorphisms of which may determine individual risk of developing urolithiasis. However, results of previous studies regarding *SPP1* polymorphisms and susceptibility to urolithiasis have apparent inconsistencies with no data available for local population.

**Methods:**

A total of 235 urolithiasis patients and 243 healthy controls, all of Pakistani ancestry, underwent genotyping for six *SPP1* genetic polymorphisms in an effort to investigate potential association with urolithiasis using indigenous candidate gene association study design. Further, a comprehensive meta-analysis following a systematic literature search was also done to ascertain an evidence based account of any existent association regarding *SPP1* promoter polymorphisms and risk of developing urolithiasis.

**Results:**

Three *SPP1* promoter polymorphisms, rs2853744:G > T, rs11730582:T > C and rs11439060:delG>G, were found to be significantly associated with risk of urolithiasis in indigenous genetic association study (OR = 3.14; *p* = 0.006, OR = 1.78; *p* = 0.006 and OR = 1.60; *p* = 0.012, respectively). We also observed a 1.68-fold positive association of a tri-allelic haplotype of these *SPP1* promoter polymorphisms (G-C-dG) with risk of urolithiasis (OR = 1.68; *p* = 0.0079). However, no association was evident when data were stratified according to gender, age at first presentation, stone recurrence, stone multiplicity, parental consanguinity and family history of urolithiasis. The overall results from meta-analysis, which included 4 studies, suggested a significant association of *SPP1* rs2853744:G > T polymorphism with susceptibility of urolithiasis (OR = 1.37; *p* = 0.004), but not for other *SPP1* polymorphic variants analyzed.

**Conclusions:**

In conclusion, we report significant association of 3 *SPP1* polymorphisms with urolithiasis for the first time from South Asia, however, this association persisted only for *SPP1* rs2853744:G > T polymorphism after meta-analysis of pooled studies. Further studies with a larger sample size will be required to validate this association and assess any potential usefulness in diagnosis and prognosis of renal stone disease.

## Background

Urolithiasis is a common urological problem (worldwide prevalence of 4–20%) [1] causing high patient morbidity and associated healthcare burden involving recurrence, frequent hospitalization and sometimes progression to renal failure resulting from chronic kidney disease (CKD) [2, 3]. Pakistan resides in the middle of Afro-Asian renal stone belt, characterized by relatively higher prevalence (12–15%) of urolithiasis, complicated by environmental determinants of urolithiasis risk such as chronic dehydration and nutrition [4].

The reported etiology of urolithiasis is multifactorial involving environmental and genetic risk factors with heritability of 50% [5]. Only a few Genome Wide Association Studies (GWAS) are available regarding urolithiasis (predominantly from European and Japanese cohorts) that identified common genetic variants in various genetic loci regulating calcium and phosphate metabolism, urinary transporters and macromolecules among others, as urolithiasis associated risk factors [6, 7]. The “common disease-common variant” paradigm stresses the small but significant risk contributed by common genetic variations in the development of complex genetic disorders like urolithiasis [8]. Although genome wide and global association analyses options are readily available and trending, still, candidate gene association studies, if executed appropriately, provide a practical and a cost effective way to evaluate genetic determinants in complex diseases, especially in research settings where resource may be limited [9].

Evidence that macromolecular proteins, especially osteopontin, may play an important role in the modulation and development of urolithiasis, is growing [10]. Osteopontin, also called as secreted phosphoprotein 1, is a macromolecular glycoprotein with pleotropic expression and function [11, 12]. In kidneys, renal epithelial cells produce osteopontin with subsequent secretion into the urine as a normal macromolecular constituent of it [13]. The hypothesis that osteopontin may play a critical role in modulating renal stone formation is supported by many observations such as; (1) SPP1 as organic component in the matrix of renal stones [14]; (2) SPP1 as important regulator of renal calcification [15]; (3) Changes in SPP1 expression and urinary SPP1 levels in hyperoxaluric rats and human subjects with urolithiasis, respectively [16]; (4) In vitro cell culture based studies and in vivo *SPP1* knockout animal models suggesting an important role of osteopontin in various phases of renal stone formation, including crystal nucleation, aggregation, retention, adhesion to renal epithelial cells and stone formation [17, 18]; and (5) Candidate gene association studies demonstrating association of *SPP1* polymorphisms and urolithiasis in different ethnic groups [19, 20].

Osteopontin gene on chromosome 4q21–25 exhibits many functional polymorphisms in the promoter/coding regions that may influence osteopontin expression/activity [21] and have been analyzed for potential association with urolithiasis in different ethnic groups [19, 20, 22, 23], but with varied results. Therefore, in this context, the current study aimed to determine any potential association between common genetic variants in osteopontin gene and the susceptibility of urolithiasis in the indigenous sample set. Further, we also applied a systematic approach by collecting and analyzing the previously available data on the osteopontin polymorphisms in association with urolithiasis susceptibility, as determined by candidate gene association studies using urolithiasis patients and healthy controls, in the form of a meta-analysis that evaluated the varying results of previous studies and provided a more comprehensive and accurate estimate of any existent association expressed as OR (95% CI) and associated *p*-value.

## Material and methods

### Case control study cohort

#### Study participants

We recruited 235 urolithiasis patients, based on ultrasound finding of at least one renal stone [supplemented by other renal stone diagnostic procedures including X-ray imaging or non-contrast-enhanced computed tomography (NCCT) and urine analysis in most cases], presenting at five different tertiary care hospitals of Punjab, during a period of 29 months. All urolithiasis patients provided clinical and pedigree details, with confirmation provided by their attending clinician (a urologist) in addition to relevant medical records, and whole blood samples (EDTA anticoagulated) for genetic study. In addition, 243 healthy subjects that were age and gender matched with same ethnic origin having no personal or familial antecedents of urolithiasis were also recruited as control group. Specific details pertaining to enrollment of study subjects and their baseline characteristics have been described earlier [24, 25].

#### Genotyping of SPP1 polymorphisms

Subjects were genotyped for six polymorphisms of *SPP1* gene including five promoter polymorphisms (rs28357094:T > G, rs11439060:delG>G, rs11730582:T > C, rs2853744:G > T and T-593A) by Sanger sequencing and one coding polymorphism (rs1126616:C > T) by PCR-RFLP based approach (assay details provided in Additional file 1). For Sanger sequencing based genotyping of *SPP1* promoter polymorphisms, two DNA fragments of 369 bp and 289 bp, covering *SPP1* promoter region, were amplified in a standard PCR reaction, purified using ethanol precipitation method and subjected to DNA sequencing. Resultant DNA sequences were aligned and analyzed using human *SPP1* reference sequence gene (NCBI accession number NG_030362.1). Two researchers independently determined the genotypes of all *SPP1* polymorphisms, based on DNA sequencing or PCR-RFLP, to minimize chances of genotyping bias.

#### Statistical analysis

The protocol followed for statistical analysis of datasets in the present study has been described elsewhere in detail [25]. Briefly, analysis of coded study data, including allelic and genotypic frequencies expressed as counts (percentages), was accomplished using the statistical package for social sciences (SPSS) version 20 for windows and online web tool SNPstats [26]. Using a Chi-square test, Hardy-Weinberg equilibrium (HWE) was performed which served as a statistical control for systematic genotyping error and population stratification where *SPP1* polymorphisms that violated HWE, as indicated by *p* < 0.05 for in the control group, were not processed for further data analyses. Odds ratios (ORs) with associated 95% confidence intervals (CI) were determined to assess strength of statistical association, if any, considering allelic, genotypic, recessive, dominant and log-additive models by the same SNPstats program. The pairwise linkage disequilibrium and haplotype analysis for *SPP1* polymorphisms was conducted using the Haploview program [27]. Bonferroni correction for multiple testing was performed in calculating the ORs and associated *p*-values for genotype and haplotype associations between *SPP1* polymorphisms and urolithiasis. Also, the *post-hoc* power of the study estimates for *SPP1* polymorphisms were performed using the Power and Sample Size Program (PS) version 3.0 available at http://biostat.mc.vanderbilt.edu/PowerSampleSize [28]. A *p* < 0.05 in two-sided analysis was considered significant unless specified differently.

### Meta-analysis of SPP1 polymorphisms and risk of urolithiasis

The current meta-analysis of selected *SPP1* genetic variants and urolithiasis susceptibility was performed using a modified protocol described in previous studies [25, 29], following the Preferred Reporting Items for Systematic Reviews and Meta-Analyses (PRISMA) guidelines [30] for performing and reporting of meta-analysis studies including considerations for literature search, eligibility, screening and selection of studies, data extraction, characteristics of included studies, reporting of effect sizes, assessment of risk of bias, summary of evidence along with any limitations, conclusions and disclosure of funding sources. A summary description of current meta-analysis protocol employed is as follows.

#### Strategy for systematic literature search

The review protocol of the present study was not pre-registered. The meta-analysis investigation included published studies using a case control study format for exploring genetic association of *SPP1* polymorphisms and urolithiasis. Initial systematic literature search identified such published studies available before September, 2018 from the online databases of the PubMed, Google Scholar, ScienceDirect, Embase and Cochrane library. The literature search used relevant keywords related to the urolithiasis (renal stones, urolithiasis, nephrolithiasis and kidney stones) and osteopontin gene polymorphisms (osteopontin, SPP1, OPN). The search was restricted to studies with human subjects only and limited to publications in English language. Additional relevant articles were included by screening the bibliography provided in the articles retrieved as a result of initial literature search. Three researchers, working independently, completed the literature search step and discrepancies were resolved through discussion.

#### Selection of studies using a defined eligibility criteria and data extraction

Selection of relevant studies for inclusion in the meta-analysis was based on the following pre-defined eligibility criteria; 1) original studies following a case-control study for determining genetic association of *SPP1* polymorphisms and urolithiasis; 2) *SPP1* polymorphic sites investigated in the study should include at least one of the mentioned sites (rs2853744, rs11730582 and rs11439060); 3) Data presentation is appropriate enabling the calculations of Odds Ratios, confidence intervals and *p*-values. In accordance with this eligibility criteria, full texts of the selected articles were used to determine the relevancy and sufficiency of the included data. To ensure the robustness of analyses performed, three researchers independently performed the screening process resolving any conflicting issues through discussion. Information about reference, publication year, region, ethnicity, total number of study subjects including number of cases and controls, source of control samples, genotyping method, Hardy-Weinberg equilibrium status and genotype frequencies of the three *SPP1* polymorphisms in cases and controls was extracted from each selected study. Collection of additional information by approaching the corresponding author of any of the included studies was not required and the data in its entirety that was included in the current meta-analysis was only extracted from published articles. The studies excluded from meta-analysis were; (1) studies with insufficient data presentation; (2) studies not pertaining to urolithiasis patients or *SPP1* polymorphisms; (3) studies not following a case-control study design; (4) review articles; (5) meta-analysis studies; (6) meeting abstracts with insufficient data; and (7) unpublished reports. The Newcastle-Ottawa scale (NOS) was the reference used to evaluate the quality of eligible studies where quality score of ≥6 was considered as threshold for inclusion of studies in the meta-analysis.

#### Statistical analysis for meta-analysis part of study

Calculations of effect sizes and, other relevant meta-analysis measures and production of visual plots (including forest and Begg’s funnel plots) was achieved using Review Manager (RevMan) version 5 [31]. Use of Mantel–Haenszel statistics enabled analysis of dichotomous /categorical data. Odds ratio (OR) with associated confidence interval (CI) represented the principal measure that reflected strength of association between *SPP1* genetic variants and urolithiasis susceptibility. The calculations of between-study heterogeneity in the current meta-analysis were based on the index of heterogeneity (*I*^*2*^) and chi-square (*χ2*) tests. A two tailed approach with a statistical significance threshold of 0.05 was used for all the statistical tests employed, except for the heterogeneity test which required the use of *p* < 0.10 as a threshold to reflect statistical significance because of the observation that the traditional Chi-square test has limited statistical power for studies involving a relatively small sample sizes. Fixed-effect model was used by default for determination of effect size, however, for studies displaying significant heterogeneity (as suggested by *I*^*2*^ values of > 50%), effect size was determined using a random effect model. The estimation of between-study variance was done using tau-squared (*τ*^*2*^) test. To assess the effect of a single study on cumulative results, each study was removed sequentially in a sensitivity analysis. Use of R version 3.5.2 [32] enabled assessment of any potential publication bias based on the Begg’s rank correlation test [33] and Egger’s linear regression test [34] . Stratified data analysis could not be performed due to limited number of studies available for meta-analysis part of the study.

## Results

### Case control study cohort

The salient demographic and clinical features of the present sample set and primary information of *SPP1* polymorphisms analyzed in this study are presented in Additional files 2 and 3, respectively. For urolithiasis patients included in this study, the median age was 34 years with a gender distribution of 1.6:1 (Males: Females ratio), both of which were comparable with that of control group (*p* = 0.77 and 0.69 for age and gender distribution, respectively). 23% of patients presented at a younger age (< 18 years). Almost half of the patients had multiple renal stones (41%), recurrent disease (49%), positive family history of urolithiasis (48%) and parental consanguinity (53%). *SPP1*–593 T/A polymorphism was found to be monomorphic in this study. The allelic and genotypic distribution for *SPP1* rs28357094:T > G and rs1126616:C > T SNPs deviated from the HWE in control group, and as a result, were not included in the subsequent data analyses. Sanger sequencing electropherograms for representative genotypes of each *SPP1* polymorphism included in final analysis are presented in Additional file 4.

Data analysis for allelic and genotypic distribution suggested no significant association between the risk of urolithiasis and any of the *SPP1* polymorphisms analyzed except for rs11439060:delG>G (OR = 0.40; *p =* 0.002 for G/dG genotype in co-dominant model) (Table 1). Additionally, *SPP1* rs2853744:G > T polymorphism showed significant associated with increased risk of urolithiasis in a dominant model (OR = 3.14; *p =* 0.006). While, *SPP1* rs11730582:T > C and rs11439060:delG>G polymorphisms were significantly associated with the risk of urolithiasis (OR = 1.78; *p =* 0.006 and OR = 1.60; *p =* 0.012, respectively) considering a recessive genetic model (Table 2).

**Table 1:** Association analysis of urolithiasis risk and *SPP1* genetic variants considering their allelic and genotypic frequencies

*SPP1* genetic variants	Genotype/Allele	Urolithiasis patients *n* = 235, n (%)	Healthy controls *n* = 243, n (%)	OR (95% CI)	*p*-value (corrected)†
rs2853744:G > T	T/T	07 (3.1%)	21 (9.1%)	Referent	0.024
	G/T	61 (27.2%)	62 (26.8%)	2.95 (1.17–7.45)	
	G/G	156 (69.6%)	148 (64.1%)	3.16 (1.31–7.66)	
	T	75 (17%)	104 (23%)	Referent	0.035
	G	373 (83%)	358 (77%)	1.44 (1.03–2.01)	
rs11730582:T > C	T/T	63 (28%)	69 (29.6%)	Referent	0.017
	T/C	87 (38.7%)	113 (48.5%)	0.84 (0.54–1.31)	
	C/C	75 (33.3%)	51 (21.9%)	1.61 (0.98–2.64)	
	T	213 (47%)	251 (54%)	Referent	0.056
	C	237 (53%)	215 (46%)	1.29 (1.00–1.68)	
rs11439060:delG > G	G/G	19 (8.3%)	12 (5%)	Referent	**0.002**
	G/dG	65 (28.5%)	103 (43.3%)	**0.40 (0.18–0.88)**	
	dG/dG	144 (63.2%)	123 (51.7%)	0.74 (0.35–1.58)	
	G	103 (23%)	127 (27%)	Referent	0.170
	dG	353 (77%)	349 (73%)	1.24 (0.92–1.68)	

†*p*-values are corrected for age and gender. *p*-value adjustment for multiple testing using Bonferroni method was also made (*p*-value threshold 0.016). Statistical significance is highlighted in bold

*OR* Odds ratio; n (%), frequency and *CI* Confidence interval

**Table 2:** Association analysis of urolithiasis risk and *SPP1* genetic variants considering dominant, recessive and log-additive genetic models

*SPP1* polymorphisms	Model	Genotypes	Patients *n* = 235, n (%)	Controls *value* 243, n (%)	OR (95% CI)	*p*-value (corrected)†
rs2853744:G > T	Dominant	T/T	07 (3.1%)	21 (9.1%)	1.00	**0.006**
		G/T-G/G	217 (96.9%)	210 (90.9%)	**3.14 (1.29–7.45)**	
	Recessive	T/T-G/T	68 (30.4%)	83 (35.1%)	1.00	0.210
		G/G	156 (69.4%)	148 (64.9%)	1.29 (0.87–1.90)	
	Log-additive	–	–	–	1.38 (1.01–1.89)S	0.040
rs11730582:T > C	Dominant	T/T	63 (28%)	69 (29.6%)	1.00	0.700
		T/C-C/C	162 (72%)	164 (70.4%)	1.08 (0.72–1.62)	
	Recessive	T/T-T/C	150 (66.7%)	182 (78.1%)	1.00	**0.006**
		C/C	75 (33.3%)	51 (21.9%)	**1.78 (1.18–2.71)**	
	Log-additive	–	–	–	1.26 (0.99–1.61)	0.062
rs11439060:delG > G	Dominant	G/G	19 (8.3%)	12 (5%)	1.00	0.150
		dG/G-dG/dG	209 (91.7%)	226 (95%)	0.58 (0.28–1.23)	
	Recessive	G/G-dG/G	84 (36.8%)	115 (48.3%)	1.00	**0.012**
		dG/dG	144 (63.2%)	123 (51.7%)	**1.60 (1.11–2.60)**	
	Log-additive	–	–	–	1.24 (0.92–1.67)	0.15

†*p*-values are corrected for age and gender. *p*-value adjustment for multiple testing using Bonferroni method was also made (*p*-value threshold 0.016). Statistical significance is highlighted in bold

*OR* Odds ratio; n (%), frequency and *CI* Confidence interval

Frequency of G-C-dG haplotype (*SPP1* rs2853744-rs11730582-rs11439060 polymorphisms, respectively) was significantly higher in urolithiasis patients as compared to controls (OR = 1.68; *p =* 0.0079), suggesting an association with increased risk of urolithiasis in haplotype analysis (Table 3). However, pair wise linkage disequilibrium (LD) and haplotype plot structure analysis demonstrated lack of any substantial LD measures (based on D’ values) for each pair of *SPP1* loci analyzed, suggesting that LD in this region is low (Additional file 5).

**Table 3:** Association of urolithiasis risk with of *SPP1* genetic variants considering haplotype analysis

*SPP1* haplotypes (rs2853744:G > T- rs11730582:T > C- rs11439060:delG > G)	Haplotype frequency†	Case, control ratios	OR (95% CI)	*p*-value (corrected)‡
G-T-dG	0.323	0.313, 0.332	Referent	–
G-C-dG	0.312	0.374, 0.248	**1.68 (1.15–2.46)**	**0.0079**
G-C-G	0.090	0.086, 0.107	0.95 (0.57–1.58)	0.840
G-T-G	0.078	0.079, 0.068	1.25 (0.61–2.56)	0.550
T-T-dG	0.060	0.083, 0.053	1.43 (0.72–2.87)	0.310
T-C-dG	0.059	0.025, 0.081	0.53 (0.24–1.17)	0.120

*OR* Odds ratio; 95% *CI* 95% confidence interval

†Haplotypes with a frequency > 5% were analyzed

‡*p*-values are corrected for age and gender. *p*-value adjustment for multiple testing using Bonferroni method was also made (*p*-value threshold 0.0083). Statistical significance is highlighted in bold

The *SPP1* polymorphisms data was also analyzed considering sub-groups of Pakistani urolithiasis patients based on demographics (gender, age at first presentation), clinical features (stone multiplicity and stone recurrence) and histories (parental consanguinity and family history of urolithiasis). But, none of these comparisons yielded any significant associations (Additional file 6).

### Meta-analysis

#### Qualitative synthesis for association of urolithiasis and SPP1 genetic variants

**Fig. 1:**
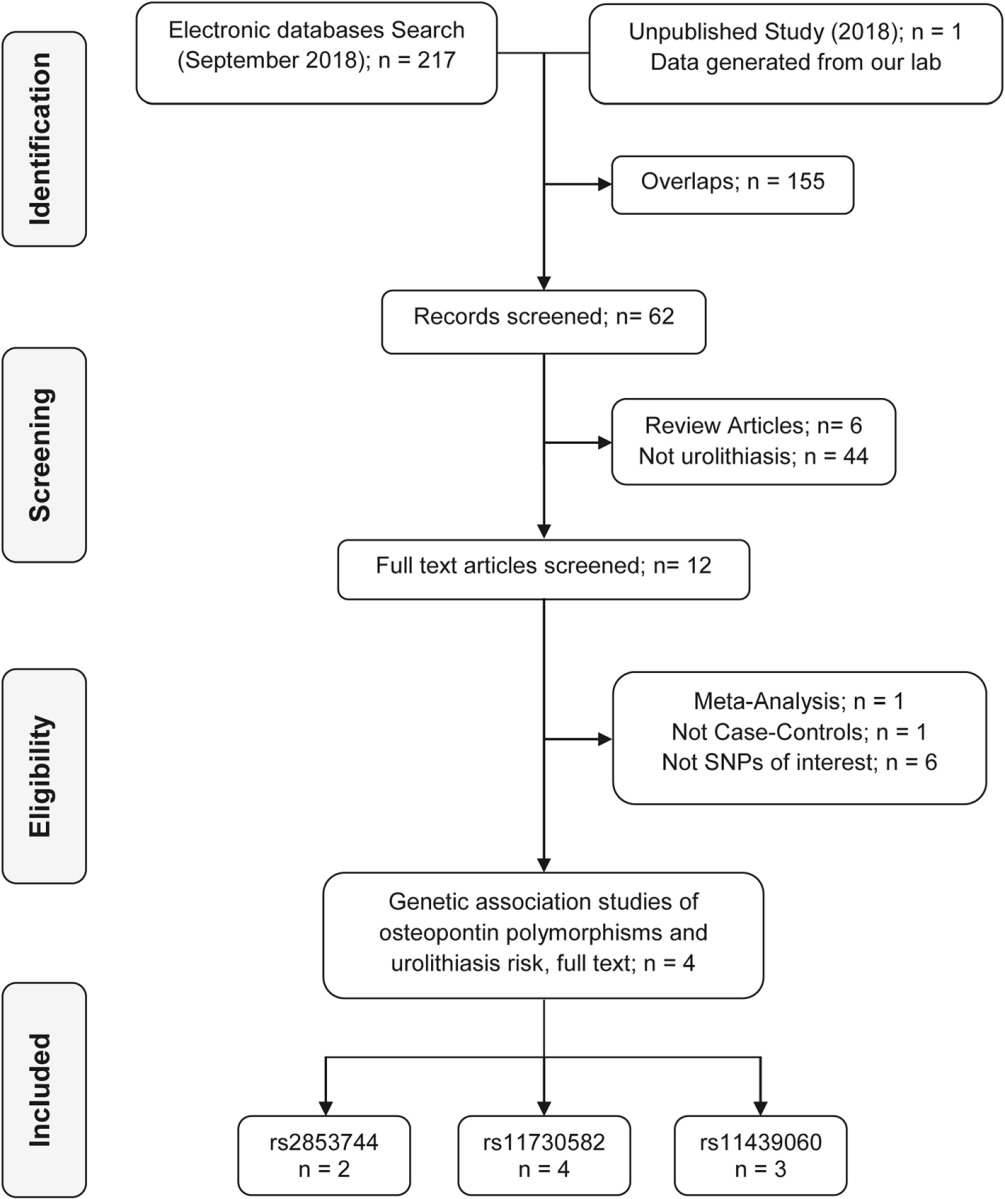
Flow diagram reflecting selection process of eligible studies included in meta-analysis. Initial literature search identified a total of 217 records from different online databases including Google Scholar, PubMed, ScienceDirect, Cochrane library and Embase. Of these, 155 overlapping studies (same studies that were indexed in two or more of these databases and resulted in redundant entries) were excluded, retaining 62 unique studies for further screening. Subsequent screening according to defined eligibility criteria yielded a total of 04 final studies including in this meta-analysis

A flow diagram, reflecting the sequence of study selection for genetic association of 3 *SPP1* genetic variants and susceptibility of urolithiasis, is described in Fig. 1. An initial online search of literature databases by means of defined MeSH terms concerning urolithiasis and osteopontin genetic variants, resulted in retrieval of a total of 217 articles. However, in the end, a total of 4 studies were included in the present meta-analysis, comprising of 3 previously published reports obtained after rigorous screening according to the eligibility criteria, combined with the indigenous genetic epidemiology study. There were 2 studies exploring the association of *SPP1* polymorphism rs2853744, 4 for rs11730582 and 3 for rs11439060. All included studies had NOS score of 6 or better.

The salient characteristics of the studies comprising the present meta-analysis are described in Table 4. The publication period for the selected studies ranged from 2010 to 2018. All four studies included in the meta-analysis were case-control studies, based on Asian population, and most (3/4) studies used control groups collected from general population. Also, all studies conformed to HWE with respect to their control group. Among these, three studies also analyzed the association of other polymorphisms in *SPP1* gene with urolithiasis. However, data pertaining to additional polymorphisms were not included in the present meta-analysis. All studies showed least one of the analyzed *SPP1* genetic variants to be positively associated with the susceptibility of urolithiasis.

**Table 4:** Main characteristics and findings of the eligible studies included in this meta-analysis

Reference (first author, year)	Region	Ethnic group	Controls source	Samples (N)	Cases	Controls	Polymorphic sites	HWE status†	Genotyping method	Findings
Liu, 2010 [35]	Taiwan	Asian	Hospital based	496	249	247	rs11730582, rs11439060	Yes	TaqMan genotyping assay	rs11439060 of *SPP1* promoter associated with risk of UL in allelic and genotypic models
Safarinejad, 2013 [19]	Iran	Asian	Population based	1026	342	684	rs2853744, rs11730582	Yes	PCR-FRET	*SPP1* SNP rs2853744 showed significant association with UL
Xiao, 2016 [20]	China	Asian	Population based	480	230	250	rs11730582, rs11439060	Yes	TaqMan genotyping assay	rs11439060 in *SPP1* promoter significantly associated with risk of UL as well as clinical characteristics in UL
Present study, 2018	Pakistan	Asian	Population based	478	235	243	rs2853744, rs11730582 and rs11439060	Yes	Sanger sequencing	All 3 *SPP1* promoter SNPs associated with UL under different genetic models

**†**Yes indicates consistence with HWE

*FRET* Fluorescence resonance energy transfer; *HWE* Hardy-Weinberg equilibrium; *N* Total number of samples; *SPP1* Osteopontin; *SNP* Single-nucleotide polymorphism; *UL* Urolithiasis

#### Quantitative synthesis for association of urolithiasis and SPP1 genetic variants

**Fig. 2:**
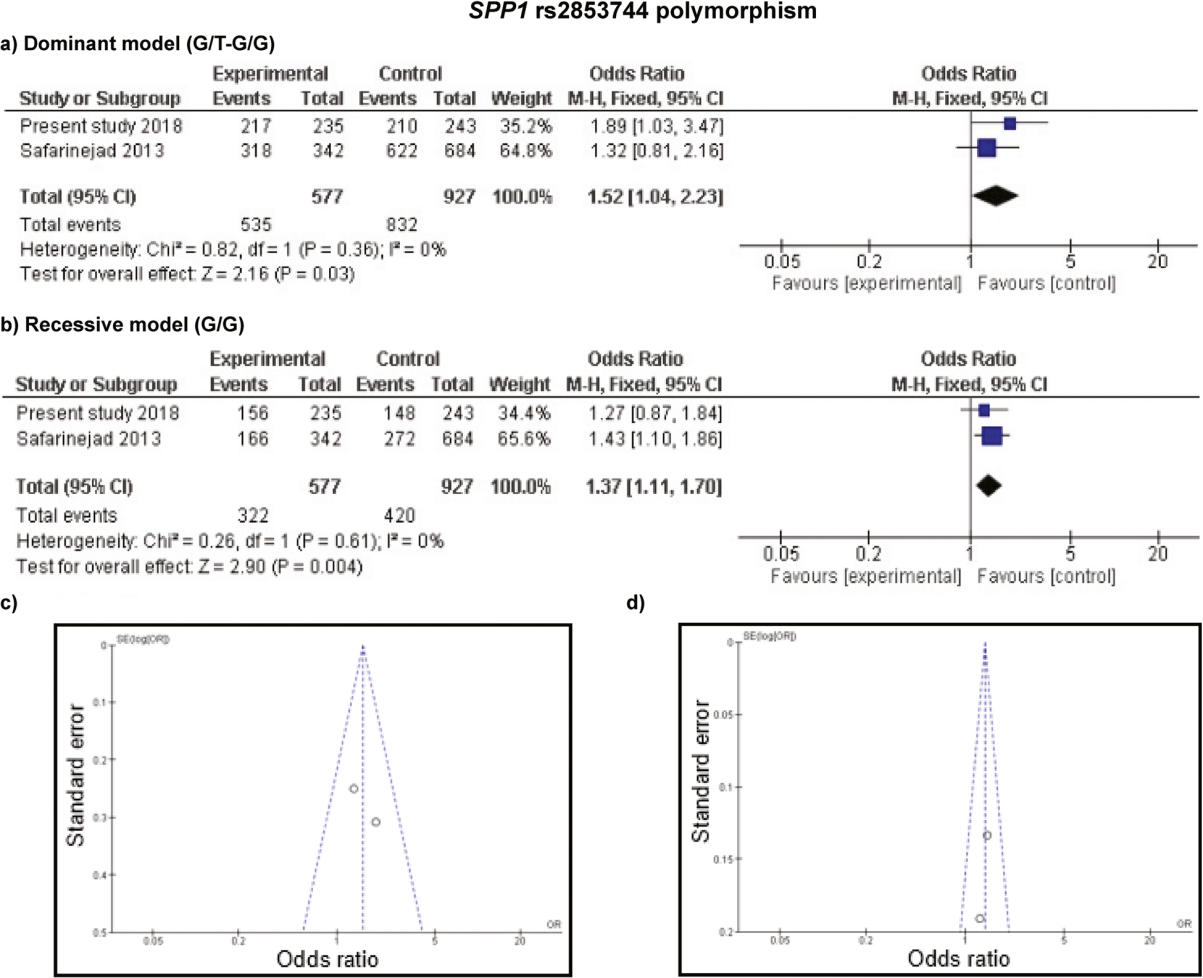
Meta-analysis of *SPP1* rs2853744:G > T polymorphism with risk of urolithiasis. **a** and **b** Forest plots of urolithiasis association with rs2853744 polymorphism assuming dominant and recessive model, respectively. **c** and **d** Funnel plots of rs2853744 polymorphism in dominant and recessive inheritance, respectively, using fixed effect model

For the association of *SPP1* rs2853744 polymorphism, only 2 studies were available including 577 cases and 927 controls where overall results from recessive model reflected a statistically noteworthy association with the susceptibility of urolithiasis (OR = 1.37; *p =* 0.004, Fig. 2b). However, no association was detected under dominant model after considering correction for multiple testing (Fig. 2a). Measures of heterogeneity in this set of studies were not significant (*I*^*2*^ = 0%, *p =* 0.61 for recessive model) therefore, fixed effect model was employed to determine the cumulative results (Fig. 2c and d).

Meta-analysis of *SPP1* rs11730582 and rs11439060 polymorphisms included 4 (1056 cases and 1424 controls) and 3 (714 cases and 740 controls) studies, respectively. The summarization of all studies indicated no significant associations between rs11730582, and rs11439060 polymorphisms and urolithiasis using either a dominant or recessive model (Additional files 7 and 8, parts a and b) after correction for multiple testing. Heterogeneity analysis for *SPP1* rs11730582 polymorphism was insignificant, but not for *SPP1* rs11439060 polymorphism, therefor fixed and random effect models, respectively, were applied in calculation of pooled results (Additional files 7 and 8, parts c and d).

Shape of funnel plots and results of Egger’s test (as depicted in Fig. 2, and Additional files 7 and 8) rendered calculation of the publication bias where absence of any significant publication bias was evident except for the analysis of *SPP1* rs11439060 polymorphism and urolithiasis (Additional file 8, parts c and d); however, the Egger’s test was not significant (*p =* 0.19 for dominant model and *p =* 0.15 for recessive model) in that case too. The outcome in sensitivity analyses, performed by removing each study at a time, indicated the reliability of the current meta-analysis results as no significant influence of an individual study was evident on the cumulative OR.

The raw dataset containing the individual phenotypic and genotypic data for each of the *SPP1* polymorphisms analyzed has been provided as additional file 9 in the supplementary data.

## Discussion

The role of genetic variations with low penetrance has earned special concern in urolithiasis research, which, in association with other risk factors, may define the critical threshold necessary for development of clinically significant renal stones. A number of genetic markers in different urolithiasis genes including *SPP1*, *VDR*, *CaSR*, urokinase, prothrombin, interleukins and others have been investigated in this regard [6]. Osteopontin has earned a particular prominence among these genetic risk factors as an importance determinant and regulator of renal calcification and stone formation [11, 13, 36]. However, the results of genetic association studies in urolithiasis are still to achieve diagnostic and translational significance. The gap in existing knowledge and inconsistent results for potential genetic associations in urolithiasis indicate a need of genetic epidemiology studies performed in diverse populations.

For the first time, we present a genetic association study investigating the role of common genetic variants in *SPP1* gene using a sample set of Pakistani patients manifesting clinically significant urolithiasis where we demonstrate significant association of three *SPP1* promoter polymorphisms (rs2853744:G > T, rs11730582:T > C and rs11439060:delG>G) with urolithiasis. We also demonstrated that subjects simultaneously harboring G-C-dG alleles of *SPP1* rs2853744-rs11730582-rs11439060 polymorphisms, respectively, have 1.68 times increased risk of urolithiasis that is statistically significant as determined by haplotype association analysis.

Moreover, estimates of *post-hoc* study power showed that the levels of power associated with *SPP1* rs2853744:G > T, rs11730582:T > C and rs11439060:delG>G polymorphisms were 78.4, 79.8 and 99.7%. These results reflect that the sample size of 235 provided fairly adequate power (almost 80%) in determining genetic associations between these polymorphic variants of *SPP1* gene and urolithiasis in the indigenous population.

We also analyzed whether additional risk factors, including gender, early age at presentation, severe disease (multiple renal stones, recurrences), presence of familial history of urolithiasis and parental consanguinity modulated the *SPP1* polymorphisms based potential genetic risk of urolithiasis. However, sub-group analyses considering these additional risk factors reflected no moderation of genetic risk for *SPP1* polymorphisms analyzed by any of the additional risk factors, at least in the context of present sample set.

Considerable heterogeneity in correlation of these *SPP1* polymorphisms and risk of urolithiasis have been reported by studies conducted in different ethnic groups. An Iranian study [19] reported a positive association of G allele/GG genotype of rs2853744:G > T with risk of developing urolithiasis. In contrast to our study results, no significant association of rs11730582:T > C with urolithiasis risk was reported in 3 independent studies from Taiwan, Iran and China [19, 20, 35]. For rs11439060:delG>G, significant association with urolithiasis phenotype was described in two studies, however, in consistence with our study, first study of Taiwanese origin reported dG/dG genotype as the risk genotype associated with increased susceptibilty of urolithiasis [35], while the second study found insertion allele or genotype (G allele or G/G genotype) to be more prevalent in Chinese urolithiasis patients as compared to controls [20].

The observed overall heterogeneity in the results of different studies regarding association of *SPP1* polymorphisms with urolithiasis may be attributable to many factors of genetic and non-genetic (including demographic, environmental and analytical variants) in origin. With respect to genetic modulators, variations in allele/genotype distributions and LD pattern of *SPP1* polymorphisms in different populations, reflecting a population specific genetic architecture, may determine substantial heterogeneity observed in genetic risk of urolithiasis. Similarly, non-genetic confounding factors may also explain a part of heterogeneity observed in the results of different genetic studies of *SPP1* polymorphisms and urolithiasis risk, including; a) differential prevalence of urolithiasis in different regions home to different ethnicities [e.g. high (12–15%) vs moderate (9.6%) vs low (5.7%) prevalence of urolithiasis in Pakistan, Taiwan and Iran, respectively] [37–39]; b) environmental risk factors (most importantly a lithogenic diet and lifestyle, and chronic dehydration) owing to differences in their context and relative contribution [4]; and c) lack of standardization and consistency in study methodologies (reflecting selection bias, control source, genotyping method used, statistical analysis approach especially when it comes to HWE conformance and applying correction for multiple testing).

Meta-analysis is a powerful tool that provides evidence based comprehensive and reliable results compared to a single study when investigating association of potential risk factors and disease phenotype. Therefore, in addition to presenting results of indigenous study, we also conducted a meta-analysis to clarify the possible association between *SPP1* polymorphisms and risk of urolithiasis. To the best of our knowledge, no meta-analysis has been carried out previously regarding association of *SPP1* promoter polymorphisms with urolithiasis risk. The results of present meta-analysis reveal that GG genotype of *SPP1* rs2853744:G > T significantly increased the risk of urolithiasis by 1.37 fold in a recessive model. However, no significant association between other *SPP1* polymorphisms analyzed (rs11730582:T > C and rs11439060:delG>G) was observed after correction for multiple testing. Several indicators of the robustness of analyses done and results generated in the current meta-analysis can be identified including; a) all the studies included in the meta-analysis were in HWE, b) no publication bias or heterogeneity was observed except for rs11439060:delG>G, c) a single study did not influenced the cumulative results as suggested by sensitivity analysis, and d) correction for multiple testing was applied. Inclusion of Pakistani dataset in the overall meta-analysis also reinforced the results obtained in this study. However, the results should still be interpreted with caution because of the limited number of primary studies available for present meta-analysis.

Currently, there is only one meta-analysis available on the subject which revealed positive association of *SPP1* coding region genetic variant (rs1126616:C > T) and lower serum and urine osteopontin levels with increased risk of developing urolithiasis [40], however, they did not include any other *SPP1* polymorphism (including *SPP1* promoter polymorphisms investigated in this study) in the analysis, which limits the usefulness and broader applicability of that study.

Despite the efforts made to generate evidence based and robust statistical results through current case control and meta-analysis study, a number of limitations should be acknowledged. First, a comprehensive investigation and correlation of biochemical parameters of renal stone disease (including stone analysis and serum/urine osteopontin levels) could not be done due to limited resources available. Second, all the eligible studies, including our own data, could not address all the known risk factors involved. Keeping in view the multifactorial nature of the urolithiasis phenotype, a more comprehensive and precise analysis should be based on adjusted estimates considering covariates such as gender, age, lifestyle, dietary habits including fluid intake, and other genetic factors, thus also investigating gene-gene and gene-environment interactions. Third, sub-group analysis based on ethnicity, source of control samples and other factors, could not be performed due to limited number of published studies available for current meta-analysis. Further, we did not include other *SPP1* polymorphisms because we could find only a couple of studies with limited sample size.

## Conclusions

In conclusion, the current study provides first account of a modest but statistically significant association of three *SPP1* promoter polymorphisms and their tri-allelic haplotype with increased risk of urolithiasis from South-Asian region under the indicated genetic model. In addition, evidence from meta-analysis part of the study supports the positive association of rs2853744:G > T *SPP1* SNP and susceptibility of urolithiasis. Further studies using larger sample sizes and analyses of gene-gene and gene-environment factors in diverse populations are suggested to validate and determine usefulness and broader relevance of *SPP1* and other genetic polymorphisms in accessing risk and prognosis of urolithiasis.

## Supplementary information

**Additional file 1. **Oligonucleotide sequences, PCR conditions and restriction enzyme used for the genotyping of *SPP1* gene polymorphisms.

**Additional file 2.** Basic characteristics of the study participants in the case-control part of the study.

**Additional file 3. **Basic information and HWE analysis for *SPP1* gene polymorphisms analyzed in this study.

**Additional file 4. **Representative electropherograms for each genotype of three *SPP1* polymorphisms. (A) rs11730582:T > C, (B) rs2853744:G > T and (C) rs11439060:delG>G.

**Additional file 5. **Pairwise linkage disequilibrium (LD) map, based on D´ values, of *SPP1* polymorphic markers analyzed in the present study. No significant LD was apparent in any of the *SPP1* polymorphic pairs analyzed.

**Additional file 6. **A Association of *SPP1* rs2853744:G > T with different clinical characteristics of urolithiasis. B Association of *SPP1* rs11730582:T > C with different clinical characteristics of urolithiasis. C Association of *SPP1* rs11439060:delG>G with different clinical characteristics of urolithiasis.

**Additional file 7. **Meta-analysis of *SPP1* rs11730582:T > C polymorphism with risk of urolithiasis. a) and b) Forest plots of urolithiasis association with rs11730582 polymorphism using dominant and recessive model, respectively. c) and d) Funnel plots of rs11730582 polymorphism assuming dominant and recessive inheritance, respectively, using fixed effect model.

**Additional file 8. **Meta-analysis of *SPP1* rs11439060:delG>G polymorphism with susceptibility of urolithiasis. a) and b) Forest plots of urolithiasis association with rs11439060 polymorphism following dominant and recessive model, respectively. c) and d) Funnel plots of rs11439060 polymorphism using dominant and recessive inheritance, respectively, by random effect model.

**Additional file 9. **Raw dataset with details of genotypes and phenotypes of every study subject analyzed for *SPP1* polymorphisms in this study.

## Data Availability

The raw sequence data used and/ analyzed in this study has been deposited in the NCBI GenBank database (https://www.ncbi.nlm.nih.gov/genbank/) and is publically available under the accession numbers MT450478 to MT450678. Sequencing data of all the cases and control samples was aligned and analyzed on the platform of *SPP1* reference sequence gene using NCBI GenBank accession number NG_030362.1 available at https://www.ncbi.nlm.nih.gov/nuccore/353249890. The datasets supporting the findings reported in this article are included in this published article and its supplementary information files. Details of individual phenotype and genotypes for each of the *SPP1* polymorphisms analyzed have been provided as additional file 9 in the supplementary data.

## Abbreviations

CaSR: Calcium sensing receptor
CI: Confidence interval
CKD: Chronic kidney disease
EDTA: Ethylenediaminetetraacetic acid
GWAS: Genome wide association studies
HWE: Hardy-weinberg equilibrium
*I*^*2*^: Index of heterogeneity test
LD: Linkage disequilibrium
MeSH: Medical subject headings
NCCT: Non-contrast-enhanced computed tomography
NOS: Newcastle-ottawa scale
OPN: Osteopontin
OR: Odds ratio
PCR-RFLP: Polymerase chain reaction-restriction fragment length polymorphism
PRISMA: Preferred reporting items for systematic reviews and meta-analyses
PS: Power and sample size program
SPP1: Secreted phosphoprotein 1
VDR: Vitamin D receptor
*τ*^*2*^: Tau-squared test
*χ2*: Chi-square test
