# Supplementary material for: Osteopontin promoter polymorphisms and risk of urolithiasis: a candidate gene association and meta-analysis study
Source: BMC Med Genet. 2020 Aug 25;21:172. doi: 10.1186/s12881-020-01101-2 (PMC7446165; doi:10.1186/s12881-020-01101-2)
Supplement: Supplementary file 1 — Additional file 1. Oligonucleotide sequences, PCR conditions and restriction enzyme used for the genotyping of SPP1 gene polymorphisms. [file 12881_2020_1101_MOESM1_ESM.docx]

**Additional file 1: Oligonucleotide sequences, PCR conditions and restriction enzyme used for the genotyping of *SPP1* gene polymorphisms**

| **Polymorphisms** | **Oligobucleotide sequence** | **Annealing temperature/Product size** | **Genotyping approach** |
| --- | --- | --- | --- |
| *SPP1* promoter region | Forward: 5’-GATAGGTAGGCTGGGCGATT-3’  Reverse: 5’-AGGTAATATCTGCAACCACTCTT-3’ | 60^o^C/369bp | DNA sequencing |
|  | Forward: 5’-TCCCTACTTTCTCCCTTTTTCA-3’  Reverse: 5’- CCAAGCCCTCCCAGAATTTA-3’ | 52^o^C/289bp |  |
| *SPP1* rs1126616:C>T | Forward: 5’-CTGAAACCCACAGCCACAAG-3’  Reverse: 5’-TCCTTACTTTTGGGGTCTACAAC-3’ | 62^o^C/175bp | PCR-RFLP assay using Alu1 restriction enzyme |

RFLP, restriction fragment length polymorphism; PCR, polymerase chain reaction; *SPP1*, osteopontin; ^o^C , centigrade and bp, base pairs.
