# Supplementary material for: Osteopontin promoter polymorphisms and risk of urolithiasis: a candidate gene association and meta-analysis study
Source: BMC Med Genet. 2020 Aug 25;21:172. doi: 10.1186/s12881-020-01101-2 (PMC7446165; doi:10.1186/s12881-020-01101-2)
Supplement: Supplementary file 2 — Additional file 2. Basic characteristics of the study participants in the case-control part of the study. [file 12881_2020_1101_MOESM2_ESM.docx]

**Additional file 2: Basic characteristics of the study participants in the case-control part of the study**

| **Characteristics** | **Cases (n = 235)** | **Controls (n = 248)** | ***p*-value** |
| --- | --- | --- | --- |
| Age [median (range), years] | 34 (2-78) | 33 (2-80) | 0.77 |
| Gender (M:F ratio) | 1.6:1 | 1.7:1 | 0.69 |
| Age at first presentation <18 years | 52/229 (23%) | - | - |
| Multiple renal stones | 95/229 (41%) | - | - |
| Recurrent urolithiasis | 113/229 (49%) | - | - |
| Parental consanguinity | 122/229 (53%) | - | - |
| Family history of urolithiasis | 110/229 (48%) | - | - |
