# Supplementary material for: Osteopontin promoter polymorphisms and risk of urolithiasis: a candidate gene association and meta-analysis study
Source: BMC Med Genet. 2020 Aug 25;21:172. doi: 10.1186/s12881-020-01101-2 (PMC7446165; doi:10.1186/s12881-020-01101-2)
Supplement: Supplementary file 3 — Additional file 3. Basic information and HWE analysis for SPP1 gene polymorphisms analyzed in this study. [file 12881_2020_1101_MOESM3_ESM.docx]

**Additional file 3: Basic information and HWE analysis for *SPP1* gene polymorphisms analyzed in this study**

| **Locus** | **SNP ID** | **Base change** | **MAF** | | ***p*-value for HWE** | **Samples typed (%)** |
| --- | --- | --- | --- | --- | --- | --- |
|  |  |  | **Cases (n = 235)** | **Controls (n = 243)** |  |  |
| –719 | rs2853744 | G/T | 0.17 | 0.23 | 0.64 | 95.2 |
| –593 | NA | T/A | 0.00^a^ | 0.00^a^ | - | 95.1 |
| –443 | rs11730582 | T/C | 0.53 | 0.46 | 0.69 | 95.8 |
| –156 | rs11439060 (previously rs17524488) | delG/G | 0.23 | 0.27 | 0.14 | 97.5 |
| –66 | rs28357094 | T/G | 0.09 | 0.09 | **< 0.0001** | 97.9 |
| c. 6982 | rs1126616 | C/T | 0.35 | 0.35 | **0.001** | 97.1 |

^a^Monomorphic polymorphism in this study

SNP ID, single-nucleotide polymorphism accession number from dbSNP NCBI (<http://www.ncbi.nlm.nih.gov/snp>; NA, not available; MAF, minor allele frequencies and HWE, Hardy–Weinberg equilibrium.
