# Supplementary material for: Osteopontin promoter polymorphisms and risk of urolithiasis: a candidate gene association and meta-analysis study
Source: BMC Med Genet. 2020 Aug 25;21:172. doi: 10.1186/s12881-020-01101-2 (PMC7446165; doi:10.1186/s12881-020-01101-2)
Supplement: Supplementary file 4 — Additional file 4. Representative electropherograms for each genotype of three SPP1 polymorphisms. (A) rs11730582:T > C, (B) rs2853744:G > T and (C) rs11439060:delG>G. [file 12881_2020_1101_MOESM4_ESM.docx]

**
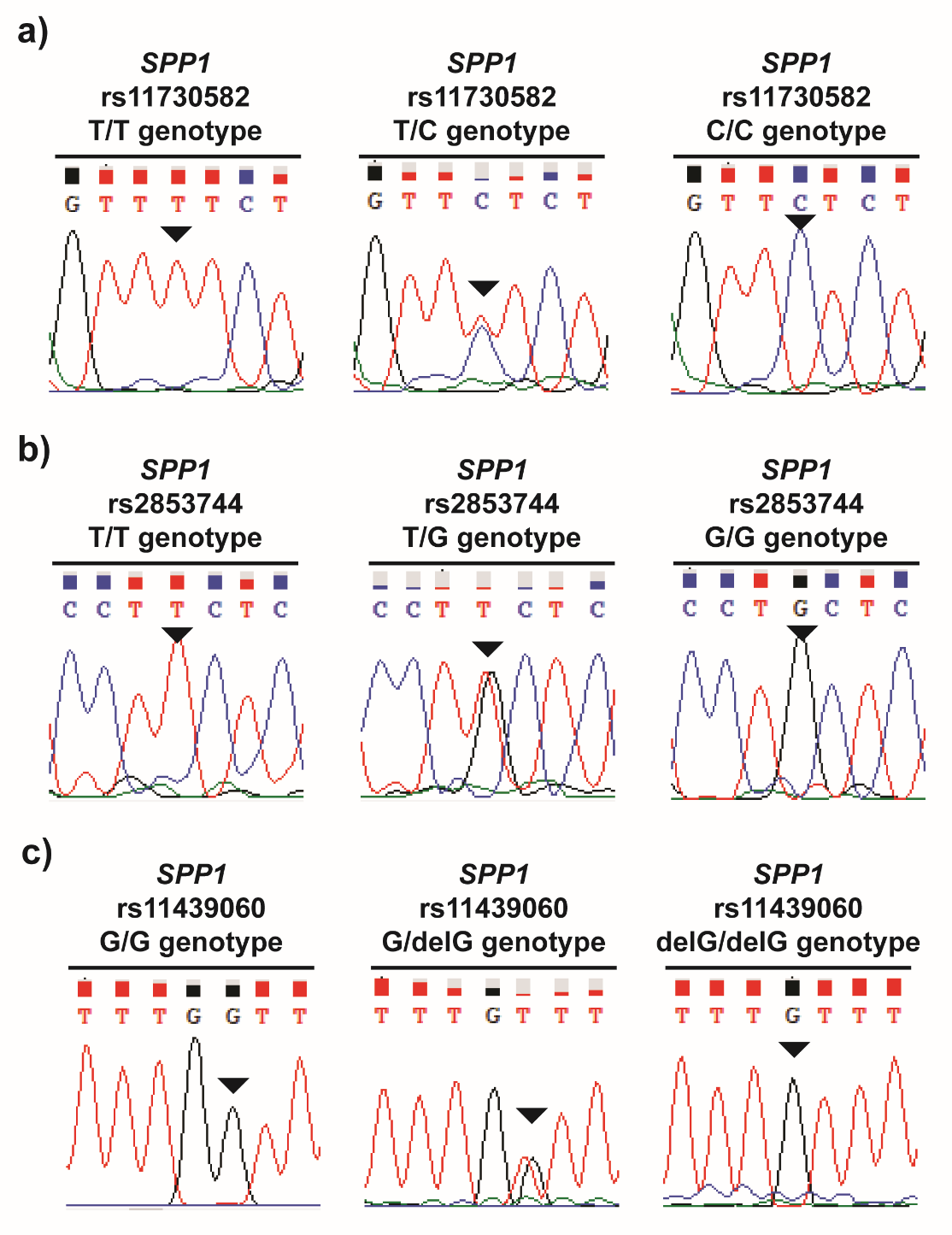
**

**Additional file 4: Representative electropherograms for each genotype of three *SPP1* polymorphisms. (A)** rs11730582:T>C, **(B)** rs2853744:G>T and **(C)** rs11439060:delG>G**.**
