# Supplementary material for: Osteopontin promoter polymorphisms and risk of urolithiasis: a candidate gene association and meta-analysis study
Source: BMC Med Genet. 2020 Aug 25;21:172. doi: 10.1186/s12881-020-01101-2 (PMC7446165; doi:10.1186/s12881-020-01101-2)
Supplement: Supplementary file 5 — Additional file 5. Pairwise linkage disequilibrium (LD) map, based on D´ values, of SPP1 polymorphic markers analyzed in the present study. No significant LD was apparent in any of the SPP1 polymorphic pairs analyzed. [file 12881_2020_1101_MOESM5_ESM.docx]

**
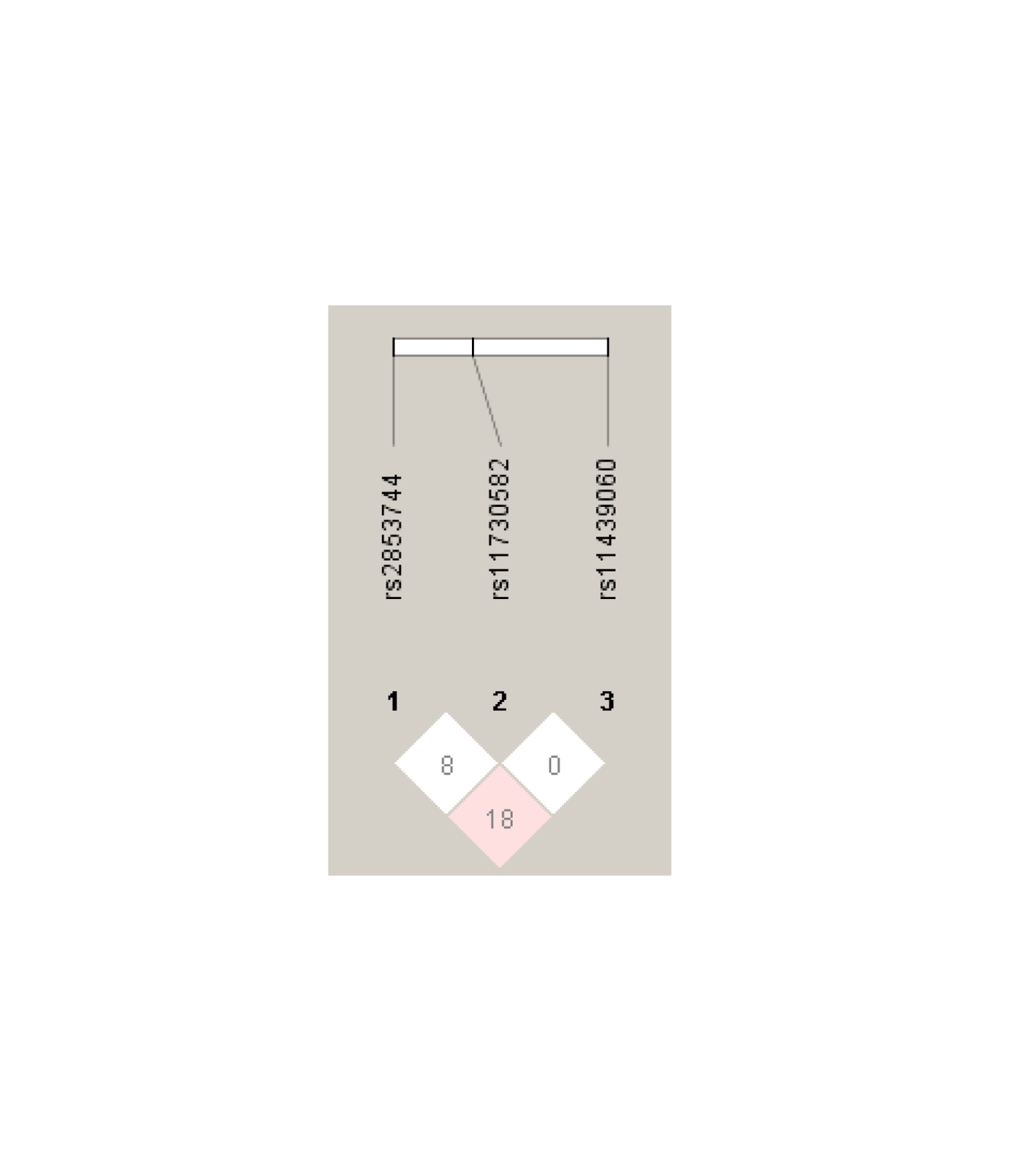
**

**Additional file 5: Pairwise linkage disequilibrium (LD) map, based on D´ values, of *SPP1* polymorphic markers analyzed in the present study.** No significant LD was apparent in any of the *SPP1* polymorphic pairs analyzed.
