# Supplementary material for: Osteopontin promoter polymorphisms and risk of urolithiasis: a candidate gene association and meta-analysis study
Source: BMC Med Genet. 2020 Aug 25;21:172. doi: 10.1186/s12881-020-01101-2 (PMC7446165; doi:10.1186/s12881-020-01101-2)
Supplement: Supplementary file 6 — Additional file 6. A Association of SPP1 rs2853744:G > T with different clinical characteristics of urolithiasis. B Association of SPP1 rs11730582:T > C with different clinical characteristics of urolithiasis. C Association of SPP1 rs11439060:delG>G with different clinical characteristics of urolithiasis. [file 12881_2020_1101_MOESM6_ESM.docx]

**Additional file 6A: Association of *SPP1* rs2853744:G>T with different clinical characteristics of urolithiasis**

| **Clinical characteristic** | ***SPP1* rs2853744:G>T (dominant model)** | | **OR (95% CI)** | ***p*-value†** |
| --- | --- | --- | --- | --- |
|  | **T/T (n, %)** | **G/T+G/G (n, %)** |  |  |
| **Gender** | | | | |
| Male | 04 (57.1%) | 129 (60%) | 1.12 (0.24-51.15) | 1 |
| Female | 03 (42.9%) | 86 (40%) |  |  |
| **Age at first presentation** | | | | |
| <18 years | 02 (28.6%) | 46 (21.7%) | 0.69 (0.13-3.68) | 0.47 |
| ≥18 years | 05 (71.4%) | 166 (78.3%) |  |  |
| **Stone recurrence** | | | | |
| Yes | 05 (71.4%) | 102 (48.1%) | 0.37 (0.07-1.95) | 0.27 |
| No | 02 (28.6%) | 110 (51.9%) |  |  |
| **Parental consanguinity** | | | | |
| Yes | 05 (71.4%) | 113 (53.3%) | 0.45 (0.08-2.40) | 0.45 |
| No | 02 (28.6%) | 99 (46.7%) |  |  |
| **Family history of urolithiasis** | | | | |
| Yes | 02 (28.6%) | 105 (49.5%) | 2.45 (0.46-12.92) | 0.44 |
| No | 05 (71.4%) | 107 (50.5%) |  |  |
| **Stone multiplicity** | | | | |
| Yes | 02 (28.6%) | 92 (43.4%) | 1.91 (0.36-10.10) | 0.47 |
| No | 05 (71.4%) | 120 (56.6%) |  |  |

†A *p*-value of <0.05 was considered statistically significant.

OR, odds ratio; CI, conﬁdence interval

**Additional file 6B: Association of *SPP1* rs11730582:T>C with different clinical characteristics of urolithiasis**

| **Clinical characteristic** | ***SPP1* rs11730582:T>C (recessive model)** | | **OR (95% CI)** | ***p*-value†** |
| --- | --- | --- | --- | --- |
|  | **T/T+T/C (n,** %**)** | **C/C (n, %)** |  |  |
| **Gender** | | | | |
| Male | 83 (55.7%) | 51 (68.9%) | 1.76 (0.97-3.17) | 0.07 |
| Female | 66 (44.3%) | 23 (31.1%) |  |  |
| **Age of onset** | | | | |
| <18 years | 31 (21%) | 18 (25%) | 1.25 (0.64-2.44) | 0.61 |
| ≥18 years | 117 (79%) | 54 (75%) |  |  |
| **Stone recurrence** | | | | |
| Yes | 67 (45.3%) | 41 (56.9%) | 1.59 (0.90-2.82) | 0.13 |
| No | 81 (54.7%) | 31 (43.1%) |  |  |
| **Parental consanguinity** | | | | |
| Yes | 81 (54.7%) | 38 (52.8%) | 0.92 (0.52-1.62) | 0.88 |
| No | 67 (45.3%) | 34 (47.2%) |  |  |
| **Family history of urolithiasis** | | | | |
| Yes | 76 (51.4%) | 32 (44.4%) | 0.75 (0.43-1.33) | 0.41 |
| No | 72 (48.6%) | 40 (55.6%) |  |  |
| **Stone multiplicity** | | | | |
| Yes | 62 (41.9%) | 32 (44.4%) | 1.10 (0.62-1.95) | 0.82 |
| No | 86 (58.1%) | 40 (55.6%) |  |  |

†A *p*-value of <0.05 was considered statistically significant.

OR, odds ratio; CI, conﬁdence interval

**Additional file 6C: Association of *SPP1* rs11439060:delG>G with different clinical characteristics of urolithiasis**

| **Clinical characteristic** | ***SPP1* rs11439060:delG>G (recessive model)** | | **OR (95% CI)** | ***p*-value†** |
| --- | --- | --- | --- | --- |
|  | **G/G+G/dG (n, %)** | **dG/dG (n, %)** |  |  |
| **Gender** | | | | |
| Male | 48 (57.1%) | 89 (62.7%) | 1.25 (0.72-2.18) | 0.49 |
| Female | 36 (42.9%) | 53 (37.3%) |  |  |
| **Age of onset** | | | | |
| <18 years | 19 (22.9%) | 31 (22.1%) | 0.95 (0.50-1.83) | 1 |
| ≥18 years | 64 (77.1%) | 109 (77.9%) |  |  |
| **Stone recurrence** | | | | |
| Yes | 37 (44.6%) | 73 (52.1%) | 1.35 (0.78-2.33) | 0.34 |
| No | 46 (55.4%) | 67 (47.9%) |  |  |
| **Parental consanguinity** | | | | |
| Yes | 47 (56.6%) | 74 (52.9%) | 0.85 (0.49-1.48) | 0.68 |
| No | 36 (43.4%) | 66 (47.1%) |  |  |
| **Family history of urolithiasis** | | | | |
| Yes | 40 (48.2%) | 69 (49.3%) | 1.04 (0.60-1.79) | 1 |
| No | 43 (51.8%) | 71 (50.7%) |  |  |
| **Stone multiplicity** | | | | |
| Yes | 30 (36.1%) | 65 (46.4%) | 1.53 (0.87-2.67) | 0.17 |
| No | 53 (63.9%) | 75 (53.6%) |  |  |

†A *p*-value of <0.05 was considered statistically significant.

OR, odds ratio; CI, conﬁdence interval
