# Supplementary material for: Osteopontin promoter polymorphisms and risk of urolithiasis: a candidate gene association and meta-analysis study
Source: BMC Med Genet. 2020 Aug 25;21:172. doi: 10.1186/s12881-020-01101-2 (PMC7446165; doi:10.1186/s12881-020-01101-2)
Supplement: Supplementary file 7 — Additional file 7. Meta-analysis of SPP1 rs11730582:T > C polymorphism with risk of urolithiasis. a) and b) Forest plots of urolithiasis association with rs11730582 polymorphism using dominant and recessive model, respectively. c) and d) Funnel plots of rs11730582 polymorphism assuming dominant and recessive inheritance, respectively, using fixed effect model. [file 12881_2020_1101_MOESM7_ESM.docx]

**
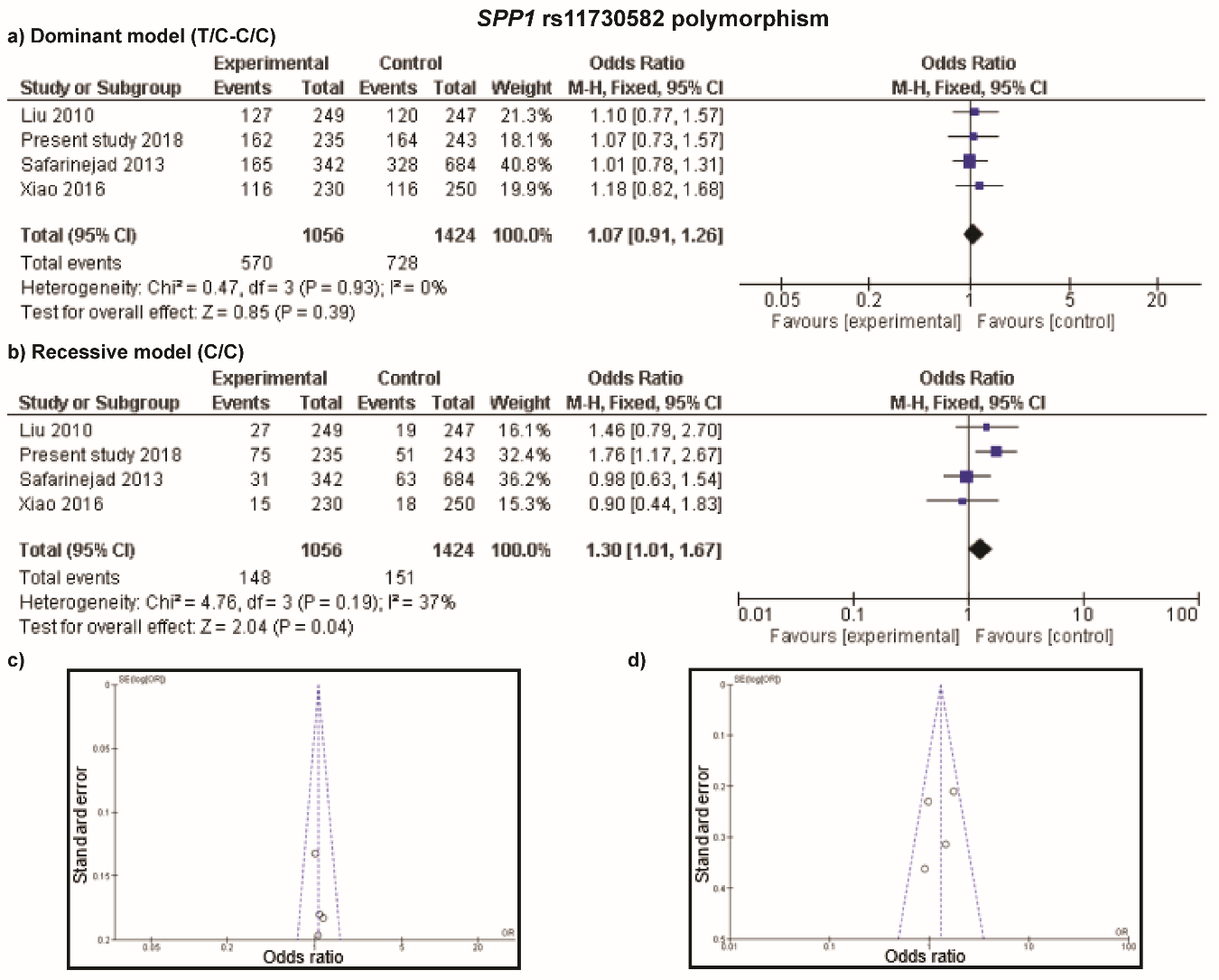
**

**Additional file 7: Meta-analysis of *SPP1* rs11730582:T>C polymorphism with risk of urolithiasis. a)** and **b)** Forest plots of urolithiasis association with rs11730582 polymorphism using dominant and recessive model, respectively. **c)** and **d)** Funnel plots of rs11730582 polymorphism assuming dominant and recessive inheritance, respectively, using fixed effect model.
