# Supplementary material for: Osteopontin promoter polymorphisms and risk of urolithiasis: a candidate gene association and meta-analysis study
Source: BMC Med Genet. 2020 Aug 25;21:172. doi: 10.1186/s12881-020-01101-2 (PMC7446165; doi:10.1186/s12881-020-01101-2)
Supplement: Supplementary file 8 — Additional file 8. Meta-analysis of SPP1 rs11439060:delG>G polymorphism with susceptibility of urolithiasis. a) and b) Forest plots of urolithiasis association with rs11439060 polymorphism following dominant and recessive model, respectively. c) and d) Funnel plots of rs11439060 polymorphism using dominant and recessive inheritance, respectively, by random effect model. [file 12881_2020_1101_MOESM8_ESM.docx]

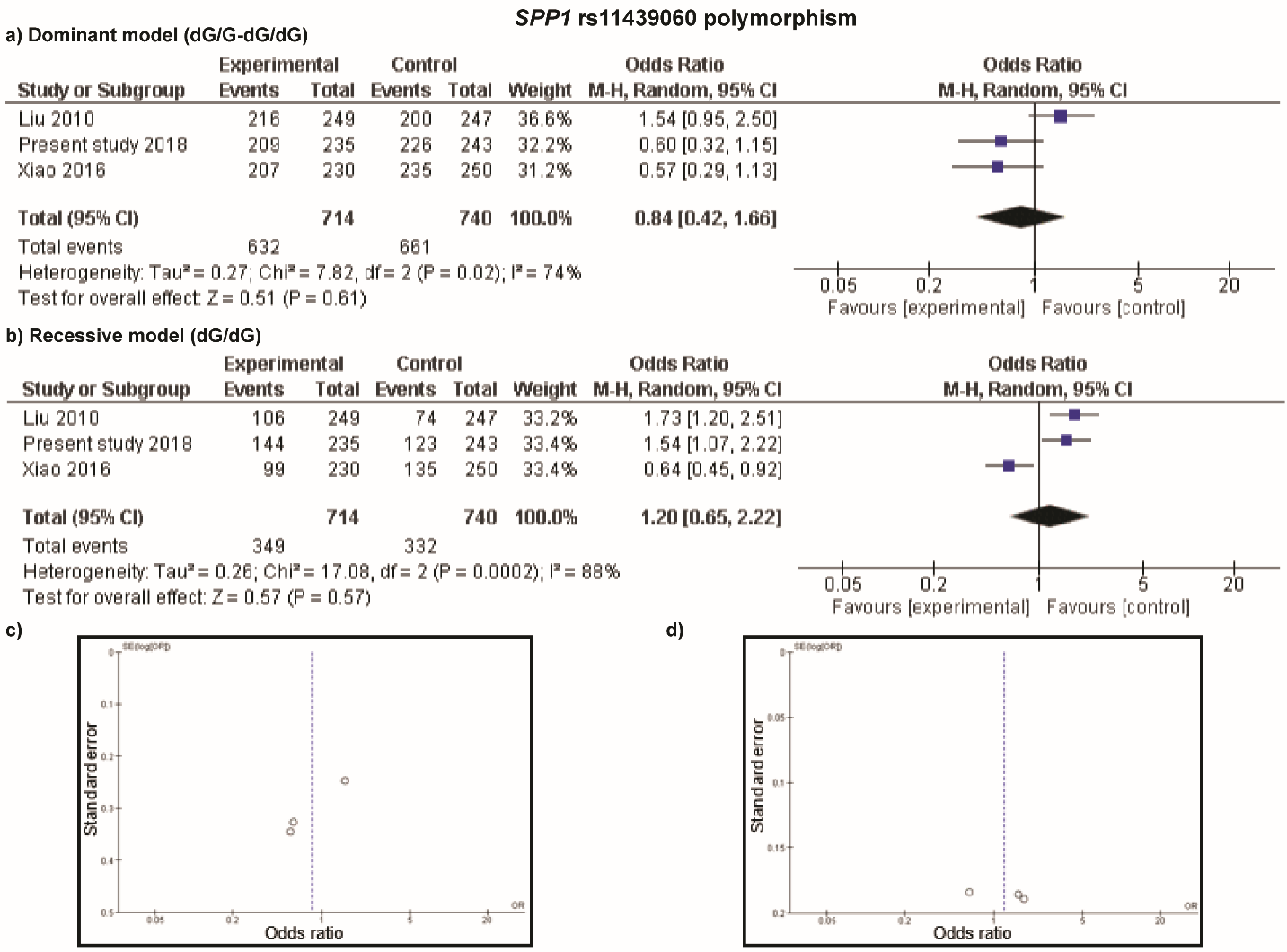


**Additional file 8: Meta-analysis of *SPP1* rs11439060:delG>G polymorphism with susceptibility of urolithiasis. a)** and **b)** Forest plots of urolithiasis association with rs11439060 polymorphism following dominant and recessive model, respectively. **c)** and **d)** Funnel plots of rs11439060 polymorphism using dominant and recessive inheritance, respectively, by random effect model.
